# Application whole exome sequencing for the clinical molecular diagnosis of patients with Duchenne muscular dystrophy; identification of four novel nonsense mutations in four unrelated Chinese DMD patients

**DOI:** 10.1002/mgg3.622

**Published:** 2019-04-01

**Authors:** Yan Zhang, Weikang Yang, Guoming Wen, Yanxia Wu, Zhiliang Jing, Dazhou Li, Minshan Tang, Guanglong Liu, Xuxuan Wei, Yan Zhong, Yanhua Li, Yongjian Deng

**Affiliations:** ^1^ Department of Pathology Shenzhen Longhua District Maternity & Child Healthcare Hospital Shenzhen P.R. China; ^2^ Department of Prevention and health care Shenzhen Longhua District Maternity & Child Healthcare Hospital Shenzhen China; ^3^ Department of Outpatient Shenzhen Longhua District Maternity and Child Healthcare Hospital Shenzhen China; ^4^ Department of Pathology Nanfang Hospital and School of Basic Medical Sciences, Southern Medical University Guangzhou P.R. China

**Keywords:** Duchenne muscular dystrophy, molecular diagnosis, nonsense mutations, novel mutations, whole exome sequencing

## Abstract

**Background:**

Duchenne muscular dystrophy (DMD) is the most common form of inherited muscular dystrophy. Germline mutations in dystrophin (*DMD*) gene cause DMD, with a X‐linked recessive mode of inheritance. Patients with DMD are usually characterized by weakness of muscle, usually started since childhood and gradually the patient will unable to stand and walk.

**Methods:**

In our present study, we identified four unrelated Chinese patients with DMD from four Chinese families. Whole exome sequencing was performed for genetic molecular analysis for these probands.

**Results:**

Whole exome sequencing and confirmatory Sanger sequencing identified four novel nonsense mutations in these four unrelated Chinese patients, respectively. All these four mutations lead to the formation of a truncated DMD protein by formation of a premature stop codon. According to the variant interpretation guidelines of American College of Medical Genetics and Genomics (ACMG), these four novel nonsense mutations are categorized as “likely pathogenic” variants.

**Conclusion:**

Our present finding not only identified four novel *loss‐of‐function* mutations in dystrophin (*DMD*) gene but also strongly emphasized the significance of whole exome sequencing as the most efficient way of identifying the candidate genes and mutations which enables us for easy and rapid clinical diagnosis, follow‐up, and management of DMD patients.

## 1 INTRODUCTION

Duchenne muscular dystrophy (DMD) [MIM# 310200] is the major form of Hereditary muscular dystrophies with a X‐linked recessive mode of inheritance (Jiang, Jiang, Xu, Shen, & Gao, 2018). DMD is manifested with severe and progressive weakness of muscles (Kyrychenko et al., 2017). DMD is the most severe and common inherited muscular dystrophy with a worldwide incidence of 1 in 3,500 to 5,000 live birth of males (Miro, Bourgeois, Claustres, Koenig, & Tuffery‐Giraud, 2018).

Germline mutations in dystrophin *(DMD)* gene cause DMD (Sardone et al., 2018). The dystrophin gene is the largest human gene, located on the short arm of the X‐chromosome. Wild‐type dystrophin gene encodes dystrophin protein of 3,685 amino acids (Tuffery‐Giraud, Miro, Koenig, & Claustres, 2017). Dystrophin protein is significantly involved in anchoring the cytoskeleton with the plasma membrane (Wein et al., 2017). Mutations in dystrophin gene causes formation of nonfunctional dystrophin protein unable to protect the muscle cells and increases the permeability of the muscle cells through which extracellular matrix enters into it and gradually causes destruction and subsequent death of muscle cells. Finally, the adipose tissues replacing the destroyed muscle cells (Zimowski, Pawelec, Purzycka, Szirkowiec, & Zaremba, 2017).

In addition, in patients with DMD, severity of the disease will not appear suddenly since the birth. As DMD is a progressive and gradually become severe, so at birth or soon after birth, most of the patients are without any clinical manifestations (McCourt et al., 2018). Moreover, clinical symptoms in DMD patients will appear around the age of 4 years (Ginsberg, McCarty, Lacomis, & Abdel‐Hamid, 2018). The disease firstly manifested with progressive proximal muscle weakness and gradually developing pseudohypertrophy of the calves (Koczok et al., 2018). In course of time, abnormal growth of the vertebrae gradually leads to the S‐shaped scoliosis (Ebrahimzadeh‐Vesal, Teymoori, Azimi‐Nezhad, & Hosseini, 2018). In most severe cases, the disease manifestations also involved failure of cardiac muscles and subsequent death (Shimo, Hosoki, Nakatsuji, Yokota, & Obika, 2018).

In recent years, development of high‐throughput sequencing technology enables us to understand the molecular genetic causes of DMD in patients with progressive proximal muscle weakness (Vieira et al., 2017). Clinical diagnosis of DMD has been done according to the clinical symptoms along with muscle biopsy, high level of serum creatine kinase (CK) enzyme, and electromyography (EMG) (Zaum et al., 2017). In our present study, we found that whole exome sequencing is the most significant and powerful way to identify the candidate mutation underlying the disease symptoms (Xu et al., 2018). Whole exome sequencing is also allowed us for timely diagnosis of DMD patients, developing effective treatment and therapies, clinical management, and genetic counselling for their families (Morikawa, Heallen, Leach, Xiao, & Martin, 2017).

## 2 MATERIALS AND METHODS

### 2.1 Ethical statement

Written informed consent was obtained from the parents. The project was approved by the ethics committee of the Department of Pathology, Nanfang Hospital and School of Basic Medical Sciences, Southern Medical University, Guangzhou 510515, China, and in accordance with the Principles of the Declaration of Helsinki.

### 2.2 Patients and pedigree

**Figure 1:**
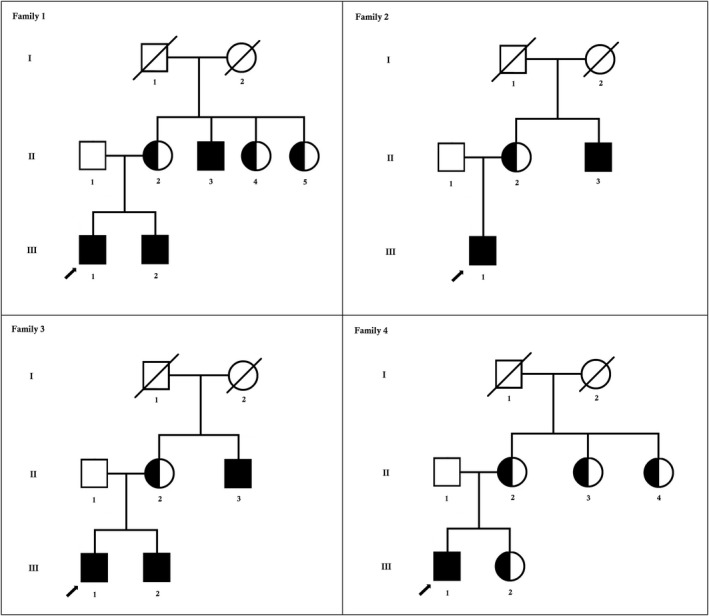
Pedigree of the family. The filled symbol indicates the patient (proband), and the half‐filled symbols show the carrier parents, who were heterozygous carriers but were unaffected. The arrow points to the proband

Here, we identified and studied four unrelated Chinese patients with progressive proximal muscular dystrophy from four unrelated Chinese families (Figure 1). Clinical diagnosis has been done in the Department of Pathology, Nanfang Hospital and School of Basic Medical Sciences, Southern Medical University, Guangzhou 510515, China.

### 2.3 Whole exome sequencing

Genomic DNA was extracted from peripheral blood using a QIAamp DNA Blood Mini Kit (Qiagen, Hilden, Germany) according to the manufacturer's instructions. All three family members (parents, proband) were subjected to exome sequencing. Sequences were captured by Agilent SureSelect version 4 (Agilent Technologies, Santa Clara, CA) according to the manufacturer's protocols. The enriched library was sequenced on an Illumina HighSeq2500. The sequencing reads were aligned to GRCh37.p10 using Burrows–Wheeler Aligner software (version 0.59). We then performed local realignment and base quality recalibration of the Burrows–Wheeler aligned reads using the GATK IndelRealigner and the GATK BaseRecalibrator, respectively (broadinstitute.org/). Single‐nucleotide variants (SNV) and small insertions or deletions (indel) were identified by the GATK UnifiedGenotyper (broadinstitute.org/). Variants were annotated using the Consensus Coding Sequences Database (20130630) at the National Center for Biotechnology Information.

**Figure 2:**
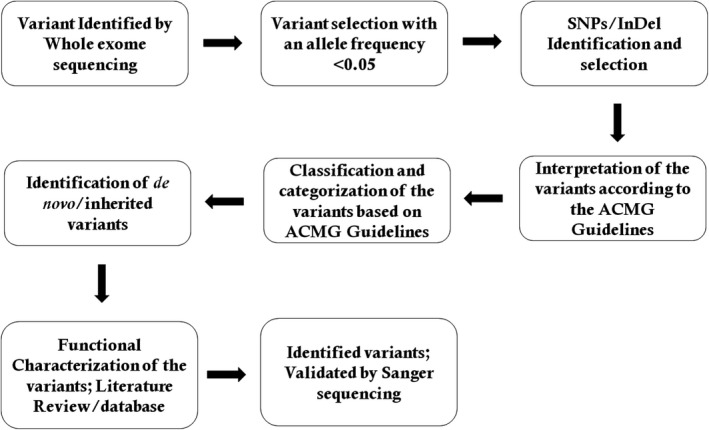
Data interpretation pipeline

We selected variations obtained from exome sequencing with minor allele frequencies <0.05 in any of the following databases (dbSNP, Hapmap, 1000 Genomes Project) and our in‐house database for ~30,000 Chinese Han samples. We also selected pathogenic and likely pathogenic variations according to the ACMG guidelines. We further compared the remaining deleterious variations in the patient with variations carried by her unaffected parents and the gene's functions with the references of OMIM and literature. The quality control details for this whole exome sequencing have been given in Table 1. Data interpretation pipeline is described in Figure 2.

**Table 1:** Whole exome sequencing Quality Control Result

Raw reads (mapped to hg19)	9459946
Raw data yield (Mb)	850.53
Reads mapped to target region	5997988
Reads mapped to flanked 100 bp region	6278988
Data mapped to target region (Mb)	488.91
Data mapped to flanked 100 bp region (Mb)	498.88
Length of target region	899883
Length of flanked 100 bp region	987909
Number of covered bases on target region	812389
Coverage of target region	99.19%
Number of covered bases on flanked 100 bp region	998709
Coverage of flanked 100 bp region	99.91%
Average sequencing depth of target region	591.98
Average sequencing depth of flanked 100 bp region	487.97

### 2.4 Sanger sequencing

To validate putative mutations, Sanger sequencing was performed. Primers flanking the candidate loci were designed based on the reference genomic sequences of the Human Genome from GenBank in NCBI and synthesized by Invitrogen, Shanghai, China. PCR amplification was carried out in an ABI 9700 Thermal Cycler. PCR products were directly sequenced on an ABI PRISM 3730 automated sequencer (Applied Biosystems, Foster City, CA). Sequence data comparisons and analysis were performed by DNASTAR SeqMan (DNASTAR, Madison, Wisconsin).

These novel mutations identified through whole exome sequencing was verified through Sanger sequencing using the following primers: F1 5′‐GACCAAGATGCTTCAGACGG‐3′, R1 5′‐GCAATTGGCCAGATTAGGA‐3′; F2 5′‐GTTCAGCAACGTCGCCAG‐3′, R2 5′‐GTTAACGGTCATCAGCGGG‐3′; F3 5′‐GTACACCGGATCGAAGGCTG‐3′, R3 5′‐GTACGCTTTGACGTCGG‐3′. The reference sequence NM_004006 of *DMD* was used.

## 3 RESULTS

### 3.1 Clinical reports

Here, we identified four Chinese probands with progressive proximal muscular dystrophy from four unrelated Chinese families. All of these probands were 10‐year‐old male from nonconsanguineous Chinese parents. These four probands presented with similar symptoms, that is, weakness of muscle in their arms, legs, and shoulders. They were unable to stand and walk. They had positive family history (Figure 1).

The proband 1 was a 10‐year‐old Chinese boy, manifested with gradual and progressive weakness of both the legs since his birth. The proband 1 has a positive family history of muscular dystrophy. We performed neurological examination and found that the power of the neck flexors is quite reduced while the power of the other muscles, that is, deltoids and bilateral hip muscles, was normal. Reflection of the muscles was progressively reduced without the involvement of neuronal sensation. Routine blood test found elevated level of muscle enzymes. In the proband 1, we found highly elevated level of several muscle enzymes, that is, CK (7,329 U/L, normal reference: <145 U/L), CK‑MB (112 U/L, normal reference: <24 U/L), alanine transaminase (152 U/L, normal reference: <45 U/L), aspartate transaminase (89 U/L, normal reference: <34 U/L), and lactate dehydrogenase (505 U/L, normal reference: <248 U/L).

In the proband 1, sinus arrythmia and left ventricular enlargement were found by electrocardiography and echocardiography. We found myogenic damage in voluntary contraction of the proband 1 by electromyography.

Patient 2 was a 10‑year‑old Chinese boy who has been suffering from gradual and progressive weakness of skeletal muscles, especially in both the legs since his birth. The proband 2 has a positive family history of muscular dystrophy. We performed neurological examination and found no abnormality in the cranial nerves. We also found that the power of both the flexor and extensor muscles in hip and knee were gradually decreasing. We found no abnormality in the sensation test and muscles tone and reflexes were quite normal. In the proband 2, we found highly elevated level of several muscle enzymes, that is, CK (6,826 U/L), CK‑MB (88 U/L), alanine transaminase (125 U/L), aspartate transaminase (74 U/L), and lactate dehydrogenase (485 U/L).

In the proband 2, mild T‐wave changes and left ventricular enlargement were found by electrocardiography and echocardiography. We found myogenic damage in voluntary contraction of the proband by electromyography.

The proband 3 was a 10‐year‐old Chinese boy, presented with slow but gradual weakness of skeletal muscles since his birth. The proband 3 also has a positive family history of muscular weakness. We performed neurological examination and found no abnormality. Muscular reflection was gradually decreasing without the involvement of neuronal sensation. In proband 3, routine blood test found highly elevated level of several muscle enzymes, that is, CK (7,167 U/L, normal reference: <145 U/L), CK‑MB (107 U/L, normal reference: <24 U/L), alanine transaminase (134 U/L, normal reference: <45 U/L), aspartate transaminase (71 U/L, normal reference: <34 U/L), and lactate dehydrogenase (498 U/L, normal reference: <248 U/L).

In the proband 3, we performed electrocardiography and echocardiography and found sinus arrythmia. Then, we performed electromyography and found myogenic damage in voluntary contraction in the proband 3.

The proband 4 was a 10‐year‐old Chinese boy, characterized by progressive loss of strength of proximal and skeletal muscles since his birth. The proband 4 also has a positive family history of muscular disorder. We performed neurological examination and found no abnormality. We found that the muscular reflections were gradually decreasing without the involvement of neuronal sensation. Routine blood test was performed and we found highly elevated level of several muscle enzymes in the proband 4, that is, CK (7,098 U/L, normal reference: <145 U/L), CK‑MB (118 U/L, normal reference: <24 U/L), alanine transaminase (143 U/L, normal reference: <45 U/L), aspartate transaminase (79 U/L, normal reference: <34 U/L), and lactate dehydrogenase (485 U/L, normal reference: <248 U/L).

In the proband 4, we performed electrocardiography and echocardiography and found no abnormality. We also found myogenic damage in voluntary contraction of the proband.

We also performed the magnetic resonance imaging (MRI) of the leg muscles and found muscular atrophy. Hence, initially, these four probands were clinically diagnosed as limb‑girdle muscular dystrophy. Then, we planned to perform whole exome sequencing for the accurate and timely diagnosis for these four probands.

### 3.2 Identification of novel mutations in *DMD* gene

**Figure 3:**
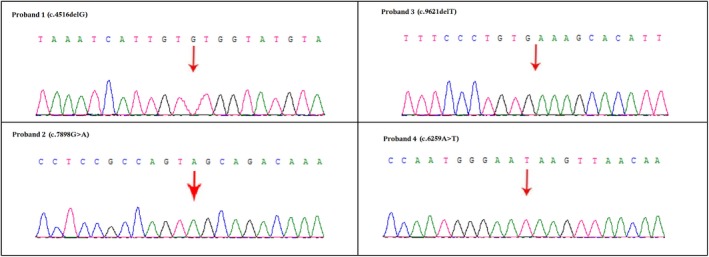
Partial DNA sequences in the Duchenne muscular dystrophy (DMD) gene by Sanger sequencing of these families

In proband 1, whole exome sequencing identified a hemizygous novel single‐nucleotide deletion (c.4516delG) in the exon 32 of the *DMD* gene (Transcript: NM_004006) (Figure 3). Sanger sequencing confirmed that this mutation well segregated among all the affected members of this family. This variant was not identified in unaffected family members as well as in 100 normal healthy control individuals. This novel hemizygous mutation leads to the formation of a premature stop codon which is predicted to produce a truncated dystrophin (p.Val1506*) protein of 1505 amino acids instead of the normal dystrophin protein with 3,685 amino acids. Hence, according to the variant interpretation guidelines of American College of Medical Genetics and Genomics (ACMG), this variant is classified as “likely pathogenic” (Richards et al., 2015). As this mutation causes the formation of a truncated protein product, so it is also called as “loss‐of‐function” mutation.

In proband 2, a hemizygous novel transition (c.7898G>A) in the exon 54 of the *DMD* gene (Transcript: NM_004006) (Figure 3). Sanger sequencing validated that this mutation is cosegregated well among affected family members. This variant was not identified in unaffected family members and also in 100 normal healthy control individuals. This novel hemizygous mutation also leads to the formation of a truncated dystrophin (p.Trp2633*) protein of 2,632 amino acids instead of the normal dystrophin protein with 3,685 amino acids. Hence, this variant is also classified as “likely pathogenic” (Richards et al., 2015). This variant is also categorized as “loss‐of‐function” mutation.

In proband 3, we identified a hemizygous novel deletion (c.9621delT) in the exon 66 of the *DMD* gene (Transcript: NM_004006) (Figure 3). Confirmatory Sanger sequencing found that this mutation is cosegregated among all the affected members of this family. This variant was not identified in unaffected family members as well as in 100 normal healthy control individuals. This novel hemizygous mutation also leads to the formation of a truncated dystrophin (p.Cys3207*) protein of 3,206 amino acids while the normal dystrophin protein is comprises of 3,685 amino acids. Hence, this variant is also classified as “likely pathogenic” variant (Richards et al., 2015). This mutation is also leading to the formation of a truncated protein product, so it is also categorized as “loss‐of‐function” mutation.

In proband 4, whole exome sequencing identified a hemizygous novel transversion (c.6259A>T) in the exon 43 of the *DMD* gene (Transcript: NM_004006) (Figure 3). Sanger sequencing revealed that this mutation is segregated well among all the affected members of this family. This variant was also not identified in unaffected family members as well as in 100 normal healthy control individuals. This novel hemizygous mutation also leads to the formation of a truncated dystrophin (p.Lys2087*) protein of 2086 amino acids while the normal dystrophin protein is comprises of 3,685 amino acids. Hence, this variant is also classified as “likely pathogenic” variant (Richards et al., 2015). This mutation is also leading to the formation of a truncated protein product, so it is also categorized as “loss‐of‐function” mutation.

These four mutations are novel and never reported before for its pathogenicity. Hence, here we reported the identification and characterization of four novel nonsense mutations in dystrophin gene in four Chinese boys with DMD for the first time.

Sanger sequencing confirmed that all of these four probands inherited these novel hemizygous variants from their mother. In addition, four mothers of these four DMD patients are heterozygous for these four mutations, respectively. The two mutations were absent in the Human Gene Mutation database (HGMD, www.hgmd.cf.ac.uk/), ExAC database, and MIM.

## 4 DISCUSSION

In the present study, we identified four novel hemizygous nonsense mutations in four unrelated Chinese patients with DMD. On the basis of the variant interpretation guidelines of American College of Medical Genetics and Genomics (ACMG), all these variants were classified as “likely pathogenic” variants (Richards et al., 2015). These four mutations were resulted in formation of four truncated dystrophin protein; hence, these four variants are *loss‐of‐function* variants.

The major function of dystrophin protein is to stabilize the muscle cells and protect them from necrosis and damage (Suzuki et al., 2016). It also plays a significant role in linking the actin microfilament with the cell membrane by the help of several anchoring proteins (Takeshita et al., 2017). So, mutation of dystrophin gene leads to formation of the nonfunctional dystrophin protein unable to protect and stabilize the muscle cells followed by developing the DMD in patients harboring dystrophin gene mutations.

However, dystrophin is the largest human gene and DMD is the most severe and common form of muscular dystrophies in China. So, genetic screening is more challenging for Chinese patients for their molecular diagnosis. The reported dystrophin gene mutations in Chinese patients are mostly large deletion and also duplication (Mccaffrey et al., 2017). Identification of the DMD patients, clinical diagnosis of those patients, understanding their family history, and possible carrier screening is the first line of therapeutic measures for the DMD families. Hence, in future, prenatal genetic screening will be very significant in order to prevent the birth of DMD patients. As there are no effective therapeutic measures for DMD, so it would be better to avoid the birth of male children with DMD. Prebirth carrier screening for the dystrophin gene mutation in pregnant mother is very significant for Chinese population.

In due course of time, due to the development of high‐throughput sequencing technology, it allows us to undertake a rapid and effective genetic screening for the DMD patients and their family members by whole exome sequencing rather than conventional Sanger sequencing. As we described earlier that the DMD is the most severe and common muscular dystrophy in China, and the dystrophin gene is the largest gene with a high rate of mutation, so Sanger sequencing of all the exons of dystrophin gene is very laborious and time‐consuming. In contrast, as muscular dystrophies are genotypically and phenotypically extremely heterozygous, so whole exome sequencing would be easier to identify the candidate genes as whole exome sequencing is allowing us to screen all the genes related to muscular dystrophies. Hence, for rapid and accurate genetic screening of the DMD patients, we recommend whole exome sequencing instead of doing conventional Sanger sequencing for the molecular diagnosis of affected individuals with DMD disease (Meregalli, Maciotta, Angeloni, & Torrente, 2016).

In addition, for better understanding of DMD disease and its prevention, medical geneticists must recommend to study the family history for a detailed pedigree analysis (Morikawa et al., 2017). Molecular genetic analysis of DMD patients with inherited dystrophin gene mutation is easier than those DMD patients with de novo mutation in dystrophin gene (Zhong et al., 2017). Therefore, carrier screening for the mother of the affected patient is very significant in order to identify whether it is an inherited case or a case of de novo mutation.

## CONFLICT OF INTEREST

The authors state no conflict of interest.

## AUTHORS’ CONTRIBUTIONS

YZ, FZ, WY, YW, ZJ, and YD designed the study and drafted the manuscript. DL, MT, GL, XW, YZh, and YL acquired and interpreted the data. All authors read and approved the final manuscript.

### Availability of data and materials

The datasets used and/or analyzed during the current study are available from the corresponding author on reasonable request.

### Ethics approval and consent to participate

The study plan and protocol have been approved by the Ethical Committee of the Department of Pathology, Nanfang Hospital and School of Basic Medical Sciences, Southern Medical University, Guangzhou 510515, China, in compliance with the Declaration of Helsinki.

### Consent for publication

Not applicable.
